# Supplementary material for: COMSUC: A web server for the identification of consensus molecular subtypes of cancer based on multiple methods and multi-omics data
Source: PLoS Comput Biol. 2021 Mar 18;17(3):e1008769. doi: 10.1371/journal.pcbi.1008769 (PMC8009357; doi:10.1371/journal.pcbi.1008769)
Supplement: S1 File — (DOCX) [file pcbi.1008769.s002.docx]

Welcome to COMSUC! This video introduces a web server for identifying consensus molecular subtypes of cancer. This project is supported by a grant from National Nature Science Foundation of China. COMSUC enables users to identify cancer consensus molecular subtypes, named CMS, by integrating multiple clustering results based on multiple platforms, multiple omics data and multiple methods. COMSUC holds the goal of clustering, fusion and interactive visualization of multi-omics data for a cohort of samples.

COMSUC is suitable for clinicians, researchers or anyone who want to discover cancer subtype.

COMSUC provides three modes for users. Firstly, users such as clinicians can upload their own patients’ data and choose one of cancer project’s data for reference to identify patients’ CMS. Secondly, users such as cancer researchers or clinicians who have a certain number of patients can upload their patients’ data alone to divide them into several consensus subgroups. Thirdly, users such as biological researchers can identify CMS of every cancer type based on one of cancer projects.

COMSUC identifies CMS by three steps. First of all, COMSUC uses different clustering methods to divide samples into different clusters. And COMSUC uses Markov Cluster algorithm to integrate clustering results producing by each single clustering method. Then, COMSUC uses Markov Cluster algorithm to integrate clustering results based on each omics data.

This is home page which contains four parts. Introduction part introduces COMSUC web server. Results box part illustrates some typical analysis results. News part lists the version history. And About part demonstates references and contact information.

This is analysis page. Take example 1 for instance. Firstly, select one of cancers listed in cancer type section. Now we select ACC. Secondly, select one of three cancer data sources or add user’s private data. Now we select TCGA project as well as upload example private file. Thirdly, select at least one type of omics data to integrate. Next, select at least one clustering method. Users can fill email for notification, but this is optional. Finally, submit the project.

The project is calculating. Users can add the URL to bookmark or record the project ID to check the results a few minutes later. Notably, analysis results can only be stored for 30 days. If a user does want to save their results for a longer time, they can contact us for other service.

Now, the calculation has been carried out. The consensus results are presented in the result page.

Firstly, check the CMS network. In this network, each node corresponds to a single subtype based on single omics data. Nodes are colored according to their CMS. Node size corresponds to the sample number in this subtype. Edge width corresponds to the Jaccard similarity coefficient. Edge transparency corresponds to –log10 P Value of Jaccard similarity coefficient. Users can change the layout and background color of CMS network. Users can also cut edges by setting a cut-off in the slide strip. Moreover, users can change other node and edge properties. Clicking a node, we can see the information of this node in the right sidebar. CMS Network are available for download in PNG and graphML formats.

Secondly, check the CMS Heatmap. Row names and column names are colored according to their CMS label. Users can change the color scheme of CMS heatmap. Clicking a block in heatmap, thus we can see the information of this block in the right sidebar.

Thirdly, check the Cross Heatmap. The heatmap shows per sample distribution across each different omics data or cluster method, grouped by the CMS and non-consensus group. Columns represent samples and rows represent single omics data or method. Unit in the heatmap represents sample subtype divided based on single omics data or by single method. By clicking the heatmap, we can see another type of cross heatmap. Columns represent samples and rows represent a subtype label divided based on single omics data or by single method.

Next, check the Sample Network. Each node represents a single sample. Network edges correspond to highly concordant subtyping calls between samples. Nodes are colored according to their CMS label, with non-consensus samples in grey. Users can interactively change the sample network as the same as the CMS network.

Then, users can check Sample Heatmap as well.

The sixth result is Survival Curve. COMSUC provides the overall P value and the P value between each pair of CMS using the Kaplan–Meier method. Users can interactively view and change the survival curve.

The seventh result is Subtype Signature. For each omics data, users can discover signatures of each CMS.

Additionally, users can view other results based on single method or single omics data by change this widget.

All results are available for downloading separately or bulk downloading.

This is Download page. Reference data including TCGA, ICGC and TARGET project are available in the first subpage Download Sample Data. All results are available in the second subpage Download Analysis Results.

For detailed information, you can switch to the manual page in COMSUC. If you have any question or suggestion, feel free to contact us by email [809848790@qq.com](mailto:809848790@qq.com).
