# Supplementary material for: COMSUC: A web server for the identification of consensus molecular subtypes of cancer based on multiple methods and multi-omics data
Source: PLoS Comput Biol. 2021 Mar 18;17(3):e1008769. doi: 10.1371/journal.pcbi.1008769 (PMC8009357; doi:10.1371/journal.pcbi.1008769)
Supplement: S2 File — (DOCX) [file pcbi.1008769.s003.docx]

Evaluation indexes of optimal cluster number

COMSUC integrates eight clustering methods, implemented by well-known and peer-recognized R packages. Some clustering method implementations have functions for choosing optimal clustering number. For theses implementations (iKmeans, iHclust, NMF, Spectralclust), COMSUC uses their own indexes of optimal clustering number chosen. And for other clustering methods, COMSUC uses four clustering evaluation indexes that are the most well-known. They are ch, db, silhouette, and dunn [1-4]. COMSUC chooses the majority of optimal cluster numbers based on four indexes.

- Ch index is Calinski-Harabasz index. It is sometimes called the variance ratio criterion (VRC), defined as

$$\mathrm{VRC}_{k}=\frac{{SS}_{B}}{{SS}_{W}}\times\frac{(N-k)}{\left( k-1 \right)}$$

where ${SS}_{B}$ is the overall between-cluster variance, ${SS}_{W}$ is the overall within-cluster variance, k is the number of clusters, and N is the number of observations. The optimal clustering solution has the biggest Calinski-Harabasz index value.

- Db index is Davies-Bouldin index. The Davies-Bouldin criterion is based on a ratio of within-cluster and between-cluster distances. The Davies-Bouldin index is defined as

$$DB=\frac{1}{k}\sum_{i=1}^{k} {max}_{j\neq i}\{D_{i,j}\}$$

where $D_{i,j}$ is the within-to-between cluster distance ratio for the *i*th and *j*th clusters. The optimal clustering solution has the smallest Davies-Bouldin index value.

- The silhouette value for each point is a measure of how similar that point is to points in its own cluster, when compared to points in other clusters. The silhouette value Si for the ith point is defined as

$$S_{i}=\frac{(b_{i}-a_{i})}{max(b_{i},a_{i})}$$

where $a_{i}$ is the average distance from the *i*th point to the other points in the same cluster as i, and $b_{i}$ is the minimum average distance from the *i*th point to points in a different cluster, minimized over clusters. The optimal clustering solution has the biggest silhouette index value.

- Dunn index is defined as

$$DI=\frac{\min_{1\leq i\leq j\leq m} \delta(C_{i},C_{j})}{\max_{1\leq k\leq m} \Delta_{k}}$$

where $\Delta_{k}$ is the size of cluster k, and $\delta(C_{i},C_{j})$ is the distance between clusters i and j. The optimal clustering solution has the biggest dunn index value.

**Reference**

1. Caliński T, Ja H. A Dendrite Method for Cluster Analysis. Communications in Statistics - Theory and Methods. 1974;3:1-27. doi: 10.1080/03610927408827101.

2. Davies DL, Bouldin DW. A Cluster Separation Measure. IEEE Transactions on Pattern Analysis and Machine Intelligence. 1979;PAMI-1(2):224-7. doi: 10.1109/TPAMI.1979.4766909.

3. Kaufman L, Rousseeuw PJ. Finding Groups in Data: An Introduction to Cluster Analysis: John Wiley; 1990.

4. Dunn JC. A Fuzzy Relative of the ISODATA Process and Its Use in Detecting Compact Well-Separated Clusters. Journal of Cybernetics. 1973;3(3):32-57. doi: 10.1080/01969727308546046.
