# Supplementary material for: COMSUC: A web server for the identification of consensus molecular subtypes of cancer based on multiple methods and multi-omics data
Source: PLoS Comput Biol. 2021 Mar 18;17(3):e1008769. doi: 10.1371/journal.pcbi.1008769 (PMC8009357; doi:10.1371/journal.pcbi.1008769)
Supplement: Attachment — Submitted filename: PCOMPBIOL-D-20-01131_review.docx [file pcbi.1008769.s004.docx]

In this manuscript the authors reported a web server, named COMSUC, used to identify the cancer Consensus Molecular Subtypes (CMSs). This tool was developed to integrate eight clustering methods and five types of omics data for 30 cancers, and was shown as being more user-friendly than previous similar tools. I noted that this lab has developed a web server ICM with integration of multi-omics data in 2016, so the current work seems to be an advance for the authors to address a need of more complex multi-omics data and more effective clustering methods. In view of the growth of omics data for cancer, I believe this work is important when the tool is extended based on more reasonable methods and designed with user-friendly operability. Herein, I have several points of concern on the manuscript and the COMSUC tool.

Major points

1. The authors emphasized several times that different clustering methods could lead to “discrepant” clustering results. This statement seems to be a little too vague. I would like to learn about what the discrepancy is among eight clustering methods? And, when they used COMSUC, to what degree did their strategy perform better (as well as being more reasonable) with integration of clustering methods? To answer these questions, perhaps some specific examples may be added into the subsection “Example use case”.

2. Moreover, in the subsection “Clustering algorithm”, the authors reported that they evaluated the optimal clustering number using different clustering performance assessment indexes. They listed these indexes according to their corresponding methods. However, I would like to learn about an important detail. How to assess the performance of a given solution of clustering in general? What about these indexes may play their roles in assessing the total performance of clustering? Maybe the authors should add a systematic analysis for this point.

3. As for multi-omics data used to analyze, it is certain that integration of omics data is helpful. The dataset contains five types of omics data from 14,954 patient samples: mRNA, miRNA, DNA methylation, copy number variation, and reverse-phase protein array (RPPA) data. The authors pointed out that the tool “… allows users to upload their own data for analysis”. Clearly it is difficult to ensure that the users’ private data are clean and good as these 14,954 patient samples. Could the authors provide an instruction of the data requirement for users’ private data? Moreover, for CMS identification, did these five types of omics data all work well? Could the authors provide a suggestion?

Minor points:

1. Page 5, Line 105, it is the first time that “MCL” appears in the text, what does “MCL” mean?

2. Page 8, Line 171-172, there are similar for “Hclust”, …, “NMF”, “SOM” etc., the authors should give their full names also. For example, “Hierarchical Clustering (Hclust),” …, “non-negative matrix factorization (NMF)”, “self-organizing maps (SOM)”

3. Page 6, Line128, should the format “.txt” be a tabular file?
