# Supplementary material for: COMSUC: A web server for the identification of consensus molecular subtypes of cancer based on multiple methods and multi-omics data
Source: PLoS Comput Biol. 2021 Mar 18;17(3):e1008769. doi: 10.1371/journal.pcbi.1008769 (PMC8009357; doi:10.1371/journal.pcbi.1008769)
Supplement: Attachment — Submitted filename: response to comment.docx [file pcbi.1008769.s005.docx]

**Point-to-point Response to Reviewers’ Comments**

**Reviewer #1:**

Comments 1:

In this manuscript the authors reported a web server, named COMSUC, used to identify the cancer Consensus Molecular Subtypes (CMSs). This tool was developed to integrate eight clustering methods and five types of omics data for 30 cancers, and was shown as being more user-friendly than previous similar tools. I noted that this lab has developed a web server ICM with integration of multi-omics data in 2016, so the current work seems to be an advance for the authors to address a need of more complex multi-omics data and more effective clustering methods. In view of the growth of omics data for cancer, I believe this work is important when the tool is extended based on more reasonable methods and designed with user-friendly operability. Herein, I have several points of concern on the manuscript and the COMSUC tool.

*Response:*

We thank the reviewer’s positive comment.

Comments 2:

The authors emphasized several times that different clustering methods could lead to “discrepant” clustering results. This statement seems to be a little too vague. I would like to learn about what the discrepancy is among eight clustering methods? And, when they used COMSUC, to what degree did their strategy perform better (as well as being more reasonable) with integration of clustering methods? To answer these questions, perhaps some specific examples may be added into the subsection “Example use case”.

*Response:*

Thanks for the detailed suggestion. According to your advice, we compared the differences between clustering results before and after consensus clustering as followings. And we have revised the corresponding parts through the manuscript (Line 263-275).

(1) Comparison between clustering results of single methods and consensus clustering method based on mRNA data of ACC. (Example 2)

The CMS heatmap illustrates that two subgroups generated by Kmeans and Hclust method are the most dissimilar. The Jaccard similarity score between these two methods is 0.828 (RFigure 1A). The cross heatmap shows that 5 non-consensus samples are divided into totally different subgroups by Kmeans and Hclust method (RFigure 1B). The P values of survival analysis generated by these five clustering methods and consensus method are listed in RTable 1. The subtype-specific signatures generated by these five clustering methods and consensus method are listed in RTable 2. It suggests that different clustering methods generate discrepant clustering results and consensus clustering method generates more significant P value of survival analysis than most of single clustering methods.

| A |  |
| --- | --- |
| B |  |

RFigure 1 The difference between subgroups of single methods and consensus clustering method based on mRNA data of ACC. (A) CMS heatmap. (B) Cross heatmap.

RTable 1 The P values of survival analysis generated by these five clustering methods and consensus method.

| Method | P Value |
| --- | --- |
| Hclust | 7.92e-4 |
| Kmeans | 1.50e-2 |
| NMF | 1.50e-2 |
| SOM | 1.30e-3 |
| Spectralclust | 3.40e-3 |
| Consensus clustering by MCL | 1.86e-3 |

RTable 2 The subtype-specific signatures generated by these five clustering methods and consensus method

| Method | Signature |
| --- | --- |
| Hclust | (18) SLPI, PLCXD3, NDRG4, SLC30A2, SGPP2, FATE1, SDK2, ETV4, C9orf84, PLD5, MC2R, C6orf176, ATP4A, PHF21B, SLC30A10, DPP10, DMKN, LMX1B |
| Kmeans | (24) SLC30A2, ABI3BP, C19orf21, LAMC2, ZNF385B, GPR115, HSD3B2, ASB4, ABCB4, ATP4A, C9orf84, ETV4, IPW, MC2R, C6orf176, LMX1B, SLC30A10, DPP10, PITX1, ZNF711, PCP4, NOV, D4S234E, CHRNA4 |
| NMF | (24) SLC30A2, ABI3BP, C19orf21, LAMC2, ZNF385B, GPR115, HSD3B2, ASB4, ABCB4, ATP4A, C9orf84, ETV4, IPW, MC2R, C6orf176, LMX1B, SLC30A10, DPP10, PITX1, ZNF711, PCP4, NOV, D4S234E, CHRNA4 |
| SOM | (12) ASB4, ATP4A, C9orf84, ETV4, D4S234E, ZNF711, MC2R, SLC30A10, DPP10, SLC30A2, ABI3BP. C19orf21 |
| Spectralclust | (21) ASB4, ATP4A, ETV4, C9orf84, D4S234E, CHRNA4, MC2R, C6orf176, PITX1, ZNF711, SLC30A10, DPP10, C19orf21, ABI3BP, SLPI, SLC7A4, ZNF385B, PPARGC1A, CPNE4, SLC30A2, SGPP2 |
| Consensus clustering by MCL | (11) SLC30A2, ASB4, ATP4A, C9orf84, ETV4, D4S234E, MC2R, DPP10, PITX1, SLC30A10, DMKN |

(2) Comparison between consensus clustering results based on three single omics levels and fusion data of ACC. (Example 1)

The CMS heatmap illustrates that two consensus subgroups generated based on mRNA and methylation data are the most dissimilar. The Jaccard similarity score between these two methods is 0.556 (RFigure 2A). The cross heatmap shows that 6 non-consensus samples are divided into totally different subgroups based on three omics data (RFigure 2B). The P values of survival analysis generated based on three omics data are listed in RTable 3. It suggests that different omics data generate discrepant clustering results and consensus clustering method generates more significant P value of survival analysis than most of individual omics data.

| A |  |
| --- | --- |
| B |  |

RFigure 2 The difference between consensus clustering results based on three single omics levels and fusion data of ACC. (A) CMS heatmap. (B) Cross heatmap.

RTable 3 The P values of survival analysis generated based on three omics data.

| Method | P Value |
| --- | --- |
| Methylation | 3.38e-3 |
| RPPA | 2.63e-3 |
| mRNA | 1.86e-3 |
| Three omics data fusion | 1.86e-3 |

Comments 3:

Moreover, in the subsection “Clustering algorithm”, the authors reported that they evaluated the optimal clustering number using different clustering performance assessment indexes. They listed these indexes according to their corresponding methods. However, I would like to learn about an important detail. How to assess the performance of a given solution of clustering in general? What about these indexes may play their roles in assessing the total performance of clustering? Maybe the authors should add a systematic analysis for this point.

*Response:*

Thanks for this suggestion. There several evaluation indexes for a given solution. COMSUC integrates eight clustering methods, implemented by well-known and peer-recognized R packages. Some clustering method implementations have functions for choosing optimal clustering number. For theses implementations (iKmeans, iHclust, NMF, Spectralclust), COMSUC uses their own indexes of optimal clustering number chosen. And for other clustering methods, COMSUC uses four clustering evaluation indexes that are the most well-known. They are ch, db, silhouette, and dunn [1-3]. COMSUC chooses the majority of optimal cluster numbers based on four indexes.

- Ch index is Calinski-Harabasz index. It is sometimes called the variance ratio criterion (VRC), defined as

$$\mathrm{VRC}_{k}=\frac{{SS}_{B}}{{SS}_{W}}\times\frac{(N-k)}{\left( k-1 \right)}$$

where ${SS}_{B}$ is the overall between-cluster variance, ${SS}_{W}$ is the overall within-cluster variance, k is the number of clusters, and N is the number of observations. The optimal clustering solution has the biggest Calinski-Harabasz index value.

- Db index is Davies-Bouldin index. The Davies-Bouldin criterion is based on a ratio of within-cluster and between-cluster distances. The Davies-Bouldin index is defined as

$$DB=\frac{1}{k}\sum_{i=1}^{k} {max}_{j\neq i}\{D_{i,j}\}$$

where $D_{i,j}$ is the within-to-between cluster distance ratio for the *i*th and *j*th clusters. The optimal clustering solution has the smallest Davies-Bouldin index value.

- The silhouette value for each point is a measure of how similar that point is to points in its own cluster, when compared to points in other clusters. The silhouette value Si for the ith point is defined as

$$S_{i}=\frac{(b_{i}-a_{i})}{max(b_{i},a_{i})}$$

where $a_{i}$ is the average distance from the *i*th point to the other points in the same cluster as i, and $b_{i}$ is the minimum average distance from the *i*th point to points in a different cluster, minimized over clusters. The optimal clustering solution has the biggest silhouette index value.

- Dunn index is defined as

$$DI=\frac{\min_{1\leq i\leq j\leq m} \delta(C_{i},C_{j})}{\max_{1\leq k\leq m} \Delta_{k}}$$

where $\Delta_{k}$ is the size of cluster k, and $\delta(C_{i},C_{j})$ is the distance between clusters i and j. The optimal clustering solution has the biggest dunn index value.

RTable 4 The impact of cluster numbers (from 2 to 7) on clustering results and final consensus clustering performance

|  | Hclust | Kmeans | NMF | SOM | Spectralclust | MCL |
| --- | --- | --- | --- | --- | --- | --- |
| P Value  (CN=2) | 7.92e-4 | 1.50e-2 | 1.50e-2 | 1.30e-3 | 3.40e-3 | 1.86e-3 |
| P Value  (CN=3) |  |  |  |  |  |  |
| P Value  (CN=4) |  |  |  |  |  |  |
| P Value  (CN=5) |  |  |  |  |  |  |
| P Value  (CN=6) |  |  |  |  |  |  |
| P Value  (CN=7) |  |  |  |  |  |  |

According to your suggestion, we use ‘Example 2’ in COMSUC as an example and pairwise P value of survival analysis as index to illustrate the impact of cluster numbers (from 2 to 7) on clustering results and final consensus clustering performance (RTable 4). In ‘Example 2’, COMSUC perform five clustering methods (Hclust, Kmeans, NMF, SOM, Spectralclust) and one consensus clustering method (MCL) based on mRNA data of ACC. Different clustering evaluation indexes all choose 2 as the optimal clustering number. RTable 4 shows that pairwise P value with 2 as clustering number is lower than the majority of those with 3 to 7 as clustering number. It suggests that these optimal clustering number assessment indexes works well and can generate proper consensus clustering result.

We have added the description of these indexes as supporting information (S2 File) in the manuscript.

Comments 4:

As for multi-omics data used to analyze, it is certain that integration of omics data is helpful. The dataset contains five types of omics data from 14,954 patient samples: mRNA, miRNA, DNA methylation, copy number variation, and reverse-phase protein array (RPPA) data. The authors pointed out that the tool “… allows users to upload their own data for analysis”. Clearly it is difficult to ensure that the users’ private data are clean and good as these 14,954 patient samples. Could the authors provide an instruction of the data requirement for users’ private data? Moreover, for CMS identification, did these five types of omics data all work well? Could the authors provide a suggestion?

*Response:*

Thank you for the detailed suggestion. For the users’ private data, there are detailed instructions in the “3.1 Mode1” of the COMSUC *Manual* page.

For each omics level and phenotype level, data format requirements are illustrated in RFigure 3.

| A |  |
| --- | --- |
| B |  |
| C |  |
| D |  |
| E |  |
| F |  |

RFigure 3 Users’ private data format requirements. (A) mRNA. (B) miRNA. (C) DNA methylation. (D) Copy Number. (E) RPPA. (F) Phenotype.

Moreover, COMSUC has the data check module to check users’ uploaded data and return error messages for unclean or bad private data. (1) If users’ omics data contains non-numeric value or NULL, COMSUC will return an error messages ‘Error! There are some non-numeric value in omics data. Please check and re-upload.’ or ‘Error! There are some NULL value in omics data. Please check and re-upload.’ (2) If users’ phenotype data contains non-numeric value, COMSUC will return an error messages ‘Error! There are some non-numeric value in some important features of clinical data. These important features include '_OS', '_OS_IND', '_EVENT', 'days_to_death', 'days_to_last_followup'. Please check and re-upload.’ (3) If number of samples in omics data and phenotype data is not equal, COMSUC will return an error messages ‘Error! Sample name across each omics data and clinical data should be the same. Please check and re-upload.’ (4) If users want to use both their private data and public data, the ratio of common features between private and public omics data should be more than 50%. Otherwise, COMSUC will return an error messages ‘Error! There are less than 50% overlap feature names between COMSUC background data and uploaded mRNA (or other omics level) data. Please check and re-upload.’

In addition, for CMS identification of different cancers, not all omics data work well. It depends on users’ data and cancer types. As a general web tool, COMSUC cannot give a clear evaluation and suggestion for each omics data. Even so, users can also explore which omics data works well for CMS identification by COMSUC. Firstly, users can integrate all omics data. COMSUC would display CMS identification results based on both multi-omics data fusion and single omics data. Then, users can evaluate which omics level works better and use it to perform next detailed analysis.

We have revised the corresponding parts (‘Data input’ section) through the manuscript (Line 205-211).

Comments 5:

Page 5, Line 105, it is the first time that “MCL” appears in the text, what does “MCL” mean?

*Response:*

We have revised the manuscript according to this suggestion (Line 179).

Comments 6:

Page 8, Line 171-172, there are similar for “Hclust”, …, “NMF”, “SOM” etc., the authors should give their full names also. For example, “Hierarchical Clustering (Hclust),” …, “non-negative matrix factorization (NMF)”, “self-organizing maps (SOM)”

*Response:*

According to this suggestion, we have added abbreviation of eight clustering methods from Line 174 to Line 177 in the manuscript.

Comments 7:

Page 6, Line128, should the format “.txt” be a tabular file?

*Response:*

The answer is yes. The format “.txt” of users’ uploaded file should be a tabular file. And we have revised the manuscript correspondingly (Line 203). Thanks again and we wish you a happy new year.

**Reference**

[1] Calinski, T., and J. Harabasz. “A dendrite method for cluster analysis.” Communications in Statistics. Vol. 3, No. 1, 1974, pp. 1–27.

[2] Davies, D. L., and D. W. Bouldin. “A Cluster Separation Measure.” IEEE Transactions on Pattern Analysis and Machine Intelligence. Vol. PAMI-1, No. 2, 1979, pp. 224–227.

[3] Kaufman L., and P. J. Rousseeuw. Finding Groups in Data: An Introduction to Cluster Analysis. Hoboken, NJ: John Wiley & Sons, Inc., 1990.

**Reviewer #2:**

Comments 1:

The manuscript present an interesting idea for a webservice for computational discovery of molecular subtypes for diverse cancers. Thera are however some concerns that preclude me to express a clear (positive) recommendation for publication.

*Response:*

Thank you for the positive comment of our idea. We have made extensive revision according to your constructive and detailed suggestions to get your positive recommendation for publication.

Comments 2:

One important concern is usability. The main purpose of the manuscript is to advertise and introduce a service. However, the web service is UNAVAILABLE at both the URL and IP addresses. Since the methods are fairly standard and the data is publicly available. User experience would be a way to establish the merit of the work. This however cannot be made with unreachable websites.

*Response:*

We are sorry for the instability of COMSUC. And we move the COMSUC to aliyun cloud server to ensure the accessibility for users around the world. The IP address of COMSUC is 59.110.25.27, and the URL is comsuc.bioinforai.tech. Moreover, we use the web speed test tool, webkaka (<http://www.webkaka.com/UrlCheck.aspx>), to test visiting time of COMSUC from 13 countries and regions (RTable 1, RFigure 1).

RTable1 Visiting time test of COMSUC from countries and regions around the world.

| IP/URL | Location | Total Time (s) | Resolution Time (s) | Download Time (s) |
| --- | --- | --- | --- | --- |
| comsuc.bioinforai.tech | USA | 1.723 | 1.261, 1.221, 1.305, 0.326 | 0.329, 0.642, 0.665, 0.985 |
|  | Germany | 2.269 | 0.237 | 2.032 |
|  | England | 1.995 | 0.230 | 1.765 |
|  | France | 2.553 | 0.221 | 2.332 |
|  | Netherlands | 1.836 | 0.226 | 1.610 |
|  | Hong Kong, China | 1.183 | 0.456, 0.110 | 0.176, 1.623 |
|  | Taiwan, China | 1.390 | 1.140 | 0.250 |
|  | Republic of Korea | 2.396 | 0.550, 1.464, 0.031, 0.590 | 5.263, 0.879, 0.185, 0.620 |
|  | Singapore | 1.541 | 0.275 | 1.266 |
|  | Japan | 5.684 | 2.086, 0.519 | 6.089, 2.673 |
|  | Malaysia | 1.908 | 1.106 | 0.802 |
|  | South Africa | 3.673 | 0.482 | 3.191 |
|  | Australian | 1.068 | 0.161 | 0.907 |
| 59.110.25.27 | USA | 0.464 | 0 | 0.328, 0.791, 0.467, 0.422 |
|  | Germany | 0.563 | 0 | 0.563 |
|  | England | 1.334 | 0 | 1.334 |
|  | France | 1.503 | 0 | 1.503 |
|  | Netherlands | 0.834 | 0 | 0.834 |
|  | Hong Kong, China | 0.099 | 0 | 0.085, 0.113 |
|  | Taiwan, China | 0.235 | 0 | 0.235 |
|  | Republic of Korea | 0.210 | 0 | 0.207, 0.276, 0.197, 0.162 |
|  | Singapore | 0.680 | 0 | 0.680 |
|  | Japan | 0.853 | 0 | 0.853 |
|  | Malaysia | 0.190 | 0 | 0.190 |
|  | South Africa | 1.852 | 0 | 1.852 |
|  | Australian | 0.332 | 0 | 0.332 |

| A |  |
| --- | --- |
| B |  |

RFigure1 Visiting time test of COMSUC from countries and regions around the world. (A) Visiting time test for URL. (B) Visiting time test for IP address.

Comments 3:

The supplementary video seems fine, but does not replace user evaluation of the tool.

*Response:*

Thanks for your positive comment, and we are sorry again for the inaccessibility of COMSUC.

Comments 4:

Aside from this, other concerns are related to documenting data processing (and pre-processing) more thoroughly since different omics call for different normalization and thresholding procedures that will impact clustering and consensus calculations. The thresholds provided "out of the blue" (disregarding sample sizes of the different databases, variability of the datasets and the different dynamic ranges of the omics) are misleading. As a quantitative biologist, I do not trust in "apparently" hand-waving arguments for calculations that have well established methods. However, a clinician may do this, precisely for this fact, solid, well-documented pre-processing methods must be provided so that quantitative biologist may explore and clinicians and experimentalist may trust the reliability of the results.

*Response:*

Thanks for this detailed and important advice.

The feature selection of clustering matrixes was filtered by choosing the most variable genes, just like used in Broad Institute GDAC Firehose Analyses Workflow Graph (<http://gdac.broadinstitute.org/Analyses-DAG.html>). For example, they generated mRNA clustering matrixes using the 1500 most variable genes (<http://gdac.broadinstitute.org/runs/analyses__latest/reports/cancer/OV/mRNAseq_Clustering_CNMF/nozzle.html>); and they generated miRNA matrixes using 647 most variable features (<http://gdac.broadinstitute.org/runs/analyses__latest/reports/cancer/OV/miRseq_Mature_Clustering_Consensus_Plus/nozzle.html>). In some conditions, this workflow graph also uses the different cutoff values in some specific matrixes. Thus, we chose 1500 most variable features in clustering matrixes of various omics data except for miRNA data (The threshold of miRNA data is 647).

To make the COMSUC both concise and informative, we hid the details of data preprocessing and threshold selection, instead of adding the threshold chosen section in the *Analysis* page. But due to our negligence, pre-processing methods were not well-documented in the previous version of manuscript. To make the data preprocessing procedure more solid, we add more description in ‘feature selection and batch effect correction’ section in the manuscript (Line 123-125).

Furthermore, we deeply agree with your opinion on threshold chosen. But we also hope that COMSUC is concise for most of users. Thus, we have added another *Download* page for users to download the data before feature selection in the latest version of COMSUC. Owe to the mode that users can upload their private data in COMSUC, users like quantitative biologists can download the data before feature selection (on *Download* page), preprocess it (in their personal computer), and upload the customized data to the COMSUC to explore CMS (on *Data Source* section of *Analysis* page). For clinicians, they can use the preprocessed data to explore CMS directly.

We added a section named “Download data and results” to emphasize the option that users can download raw data, preprocess it by themselves, and upload as private data in COMSUC to perform the same analysis. Thanks again for this constructive and inspired suggestion (Line 244-253).

Comments 5:

It will be interesting to document also why did the authors choose the MCL algorithm over competing alternatives. A solid rationale, perhaps supported by benchmark tests is desirable.

*Response:*

Thank you for the suggestion. In the previous version, the reason why we choose the MCL algorithm is stated as followings. 1) MCL algorithm is a graph-based clustering method. For the cross matrix recording clustering results based on single methods, MCL algorithm can identify non-consensus clustering groups based on single methods, while other methods cannot. 2) MCL has been successfully applied to identify consensus molecular subtypes of colorectal cancer in the Guinney’s research [1]. He integrated six single clustering methods using MCL and four consensus molecular subtypes and one non-consensus (or mixed) subtypes. This research inspired us to design a more user-friendly computational tools for identifying consensus molecular subtypes of cancer.

Inspired by your suggestions about this and last question, we agree with the idea that COMSUC should provide more choice for different users. Thus, we have added two more consensus clustering methods, COCA (Cluster-of-Cluster Assignments) and SuperCluster, in the latest version of COMSUC. COCA is originally developed to define subgroups in the TCGA breast cancer cohort and has been successfully applied to identify pan-cancer groups across 12 cancer types based on multi-omics data [2,3]. SuperCluster is originally developed to define subclasses in the TCGA endometrial carcinoma and also has been successfully applied to identify pan-cancer groups across 12 cancer types based on multi-omics data [3,4]. We apply the same function of Guinney’s research [1] in COCA and SuperCluster algorithms to identify non-consensus samples, but the two algorithms also have no ability to identify non-consensus clustering groups based on single methods.

To illustrate the similarities and differences between MCL and the two added methods, we tested them across several cancers, single clustering methods, and omics levels (RTable 2). It suggests that MCL tends to generate clearer CMS network, more non-consensus groups, and more significant P Value of survival analysis.

We have revised the manuscript and website accordingly.

RTable 2 Comparison of three consensus clustering methods across several cancers, clustering method, and omics levels

| Cancer | Omics Level | Cluster Method | Consensus Method | Non-consensus Samples | CMS Network | Survival P Value | Task ID |
| --- | --- | --- | --- | --- | --- | --- | --- |
| ACC  TCGA | Methylation RPPA mRNA | Hclust Kmeans NMF SOM Spectralclust | MCL | 6 |  | 1.86e-3 | H26C1ynE3  (Example 1) |
| ACC  TCGA | Methylation RPPA mRNA | Hclust Kmeans NMF SOM Spectralclust | COCA | - (RPPA data generates only one CMS) | - | - | f_fCvD09y  (rrtKdZ04n) |
| ACC  TCGA | Methylation RPPA mRNA | Hclust Kmeans NMF SOM Spectralclust | SuperCluster | 7 |  | 2.54e-3 | h0xgZcRw6 |
| ACC  TCGA | mRNA | Hclust Kmeans NMF SOM Spectralclust | MCL | 5 |  | 1.86e-3 | dy6K_UBVW  (Example 2) |
| ACC  TCGA | mRNA | Hclust Kmeans NMF SOM Spectralclust | COCA | 7 |  | 2.26e-3 | 647oL9kc6 |
| ACC  TCGA | mRNA | Hclust Kmeans NMF SOM Spectralclust | SuperCluster | 7 |  | 2.26e-3 | iL0XscyuL |
| KIRC  TCGA | mRNA | APclust Hclust Kmeans NMF SOM Spectralclust iHclust iKmeans | MCL | 12 |  | 3.26e-2 | uwO1VRffj |
| KIRC  TCGA | mRNA | APclust Hclust Kmeans NMF SOM Spectralclust iHclust iKmeans | COCA | 22 |  | 7.27e-2 | aAfei@4FR |
| KIRC  TCGA | mRNA | APclust Hclust Kmeans NMF SOM Spectralclust iHclust iKmeans | SuperCluster | 12 |  | 3.26e-2 | 2PjjPFrsz |
| BLCA  ICGC | mRNA | APclust Hclust Kmeans NMF SOM Spectralclust iHclust iKmeans | MCL | 40 |  | 4.06e-2 | CUK9YW3Li |
| BLCA  ICGC | mRNA | APclust Hclust Kmeans NMF SOM Spectralclust iHclust iKmeans | COCA | 45 |  | 1.85e-2 | eTV347mBi |
| BLCA  ICGC | mRNA | APclust Hclust Kmeans NMF SOM Spectralclust iHclust iKmeans | SuperCluster | 40 |  | 4.06e-2 | Xm8N4ZA4c |

Comments 6:

Once these concerns have been addressed, I may express a more favorable opinion on this work.

*Response:*

We have added more functions and revised the webserver and the manuscript according to your constructive suggestions. Thanks again and we wish you a happy new year.

**Reference:**

[1] Guinney J, Dienstmann R, Wang X, de Reynies A, Schlicker A, Soneson C, et al. The consensus molecular subtypes of colorectal cancer. Nat Med. 2015; 21: 1350-1356.

[2] Hoadley K A, Yau C, Wolf D M, et al. Multiplatform analysis of 12 cancer types reveals molecular classification within and across tissues of origin[J]. Cell, 2014, 158(4): 929-944.

[3] Cancer Genome Atlas Network. Comprehensive molecular portraits of human breast tumours[J]. Nature, 2012, 490(7418): 61.

[4] Levine D A, Cancer Genome Atlas Research Network. Integrated genomic characterization of endometrial carcinoma[J]. Nature, 2013, 497(7447): 67-73.
